# Maternal Benzene Exposure during Pregnancy and Risk of Childhood Acute Lymphoblastic Leukemia: A Meta-Analysis of Epidemiologic Studies

**DOI:** 10.1371/journal.pone.0110466

**Published:** 2014-10-15

**Authors:** Yanfeng Zhou, Shaozun Zhang, Zhen Li, Jie Zhu, Yongyi Bi, YuE Bai, Hong Wang

**Affiliations:** Department of Occupational and Environmental Health, School of Public Health, Wuhan University, Wuhan, P. R. China; East Carolina University, United States of America

## Abstract

**Background:**

The prevalence of childhood leukemia is increasing rapidly all over the world. However, studies on maternal benzene exposure during pregnancy and childhood acute lymphoblastic leukemia (ALL) have not been systematically assessed. Therefore, we performed a meta-analysis to investigate the association between maternal solvent, paint, petroleum exposure, and smoking during pregnancy and risk of childhood ALL.

**Methods:**

Relevant studies up to September 1^st^, 2013 were identified by searching the PubMed, EMBASE, Cochrane library and the Web of Science databases. The effects were pooled using either fixed or random effect models based on the heterogeneity of the studies.

**Results:**

Twenty-eight case-control studies and one cohort study were included for analysis, with a total of 16,695 cases and 1,472,786 controls involved. Pooled odds ratio (OR) with 95% confidence interval (CI) for ALL was 1.25 (1.09, 1.45) for solvent, 1.23 (1.02, 1.47) for paint, 1.42 (1.10, 1.84) for petroleum exposure, and 0.99 (0.93, 1.06) for maternal smoking during pregnancy. No publication bias was found in this meta-analysis and consistent results were observed for subgroup and sensitivity analyses.

**Conclusions:**

Childhood ALL was associated with maternal solvent, paint, and petroleum exposure during pregnancy. No association was found between ALL and maternal smoking during pregnancy. Avoidance of maternal occupational and environmental benzene exposure during pregnancy could contribute to a decrease in the risk of childhood ALL.

## Introduction

In recent years, leukemia has become the most common cancer of children under the age of 15 [1]. About 75%–80% of leukemia cases are acute lymphoblastic leukemia (ALL) [2],[3], which is five times more prevalent than acute myelogenous leukemia (AML) [4]–[8]. The average annual incidence rate of childhood leukemia per million is 16.4 (standard deviation [SD] 13.6) in low-income countries, 36.5 (SD 11.6) in middle-income countries, and 40.9 (SD 6.1) in high-income countries [9]. The incidence of leukemia has been rising rapidly among children under 5 years old [10], [11], while declining among children aged 5–19 years [12]. A number of possible risk factors (e.g., infectious, genetic, or environmental) have been explored in an attempt to determine the etiology of childhood leukemia. To date, some risk factors for ALL including ionizing radiation, specific chromosomal and genetic abnormalities [1], [13], [14] have been well documented. The association between ALL and other risk factors, such as infectious agents, environmental factors, drug use, and chemical exposure are still unclear. [8], [14]–[16].

In previous studies, benzene has been identified as a carcinogen for leukemia among exposed workers [17]. However, occupational exposure to benzene directly in children has never been reported. Recently, several epidemiological studies have indicated that increasingly environmental benzene exposure is potentially a major cause of childhood leukemia. For example, Freedman et al. [18] have identified elevated risk among children whose mothers were exposed to interior house painting during the year before the children’s birth. Moreover, experimental research had demonstrated the cytotoxicity of hydroquinone on yolk sac hematopoietic stem cells was more noticeable than on bone marrow hematopoietic stem cells [19]. Therefore, it is a reasonable assumption that childhood leukemia may have occurred during the fetal stage of development. Accordingly, maternal exposure to leukemogenic factors in early pregnancy may increase hematopoietic system DNA instability, genetic susceptibility to cancer, and oncogenic lesions of the hematopoietic system during fetal stage, which ultimately results in the development of childhood leukemia in their offspring [9].

Among environmental risk factors, the impact of solvent, paint, petroleum products and cigarette smoking on the development of childhood ALL have been extensively investigated. Studies have shown that cigarette smoking is leading contributor of benzene in non-occupational environments [20]–[23]. Cigarettes contains a variety of carcinogenic substances including benzene and butadiene, and there are 48 µg benzene and 38.5 µg butadiene per cigarette [24]. Therefore, pregnancies exposed to the benzene are very common, particularly among pregnant mother who smoke. With regard to childhood ALL, some studies revealed a significant positive association whereas an inverse association was reported by others. The aim of our study was to perform a meta-analysis of cohort and case-control studies in a comprehensive overview of all available knowledge to provide a more thorough mathematical assessment summarizing the possible relationships between maternal solvent, paint, petroleum exposure and smoking during pregnancy and childhood ALL.

## Materials and Methods

### Search strategy

A comprehensive review of the literature was conducted by searching PubMed, Embase, the Cochrane library and Web of Science databases up to September 1^st^ 2013. The search terms were: (childhood OR child OR infant) AND (leukemia OR cancer OR malignancy) AND (environmental factor OR occupational exposure OR benzene OR hydroquinone OR smoking OR paint OR petroleum OR solvent). Search strategies are shown in Table 1. Additionally, we carried out a manual search using reference lists of retrieved original articles and recent reviews. However, we did not search the grey literature.

**Table 1:** Search Strategy for PubMed (up to September 1^st^, 2013).

Search strategy	Search terms
#1	childhood
#2	child
#3	infant
#4 #1 OR #2 OR #3	
#5	leukemia
#6	cancer
#7	malignancy
#8 #5 OR #6 OR #7	
#9	Environmental factors
#10	occupational exposure
#11	benzene
#12	hydroquinone
#13	smoking
#14	paint
#15	petroleum
#16	solvent
#17 #9 OR #10 OR #11 OR #12 OR #13 OR #14 OR #15 OR #16	
#18 #4 AND #8 AND #17	

### Study selection criteria

Studies were selected for inclusion based on the following criteria: 1) the information to estimate the relationship between benzene exposure and risk for childhood leukemia (effect size) in terms of odds ratio (OR), relative risk (RR) and hazard risk (HR) was available; 2) the study examined maternal exposure during pregnancy; 3) the study design was a cohort or case-control study; 4) current smoking during pregnancy was included; and 5) studies with children who had Down’s syndrome or any neoplastic disease were excluded. When multiple publications on the same study population were identified or study populations overlapped, the most recent and complete study was included in the meta-analysis. Furthermore, if a variable representing “any” solvent or paint or petroleum exposure was reported, these data were used. Only studies published in English were included. For reliability,two co-authors (Zhou and Zhang) reviewed all the identified citations independently, and articles met the criteria of inclusion were finally included in the present meta-analysis. Disagreements were resolved by reaching consensus between the two co-authors.

### Data extraction and quality assessment

Information from studies was also extracted independently by two researchers, with disagreements resolved by consensus. The following terms of data were collected: first author’s last name, country studied, study design, study period, age range, matching factors, adjusting factors, exposure definition, the number of cases and controls, effect estimate (OR, RR, HR) with 95% CIs, and assessment of exposure. Most of the time, the effect estimate were extracted from the papers directly, however, the ORs were calculated when the paper only reported frequencies. All literatures analyzed in this meta-analysis are publicly published and are accessible in the database using the citation. The data for each literature are extracted using designed standard data extract form, which are available to all readers as required.

The Newcastle-Ottawa-Scale (NOS) was used to assess the quality of papers [25]. The NOS for cohort and case-control studies includes the following items: 1) representativeness of the exposed cohort/adequacy of case definition; 2) selection of the non-exposed cohort/representativeness of the cases; 3) ascertainment of exposure/selection of controls; 4) demonstration that outcome of interest was not present at start of study/definition of controls; 5) comparability of cohorts on the basis of the design or analysis/comparability of cases and controls on the basis of the design or analysis; 6) assessment of outcome/ascertainment of exposure; 7) sufficiency of follow-up for outcomes to occur/similarity of method of ascertainment for cases and controls; and 8) adequacy of follow-up of cohorts/non-response rate. The full score was 9 stars, and studies with scores of 0–3, 4–6, 7–9 were considered as low, moderate and high quality, respectively. Quality assessment was independently extracted by two co-authors, and any disagreements were resolved by consensus.

### Statistical analysis

Meta-analysis was performed using Stata version 12.0 (Stata Corporation, College Station, Texas). ORs, RRs, and HRs with 95% corresponding CIs were used (HR and RR were directly considered as OR) to assess the association between benzene exposure and risk for childhood ALL. Heterogeneity among studies was estimated by chi-squared test and Cochran Q score (reported as *I^2^*) with corresponding *P*-values and the level of significance was set at *P* = 0.10[26]. If *P*<0.10 or *I^2^*≥50%, the heterogeneity was considered statistically significant, and the DerSimonian and Laird random-effect model was used; Otherwise, the Mantel-Haenszel fixed-effect model was used to calculate the pooled ORs [27].

Publication bias was assessed using the STATA procedure of ‘Metabias’, which is based on two different approaches, Begg funnel plots [28] and Egger’s tests [29]. Moreover, we conducted sensitivity analyses by sequential omission of individual studies under various contrasts to reflect the influence of the individual data to the pooled ORs and evaluate the stability of the results.

## Results

### Literature search

**Figure 1:**
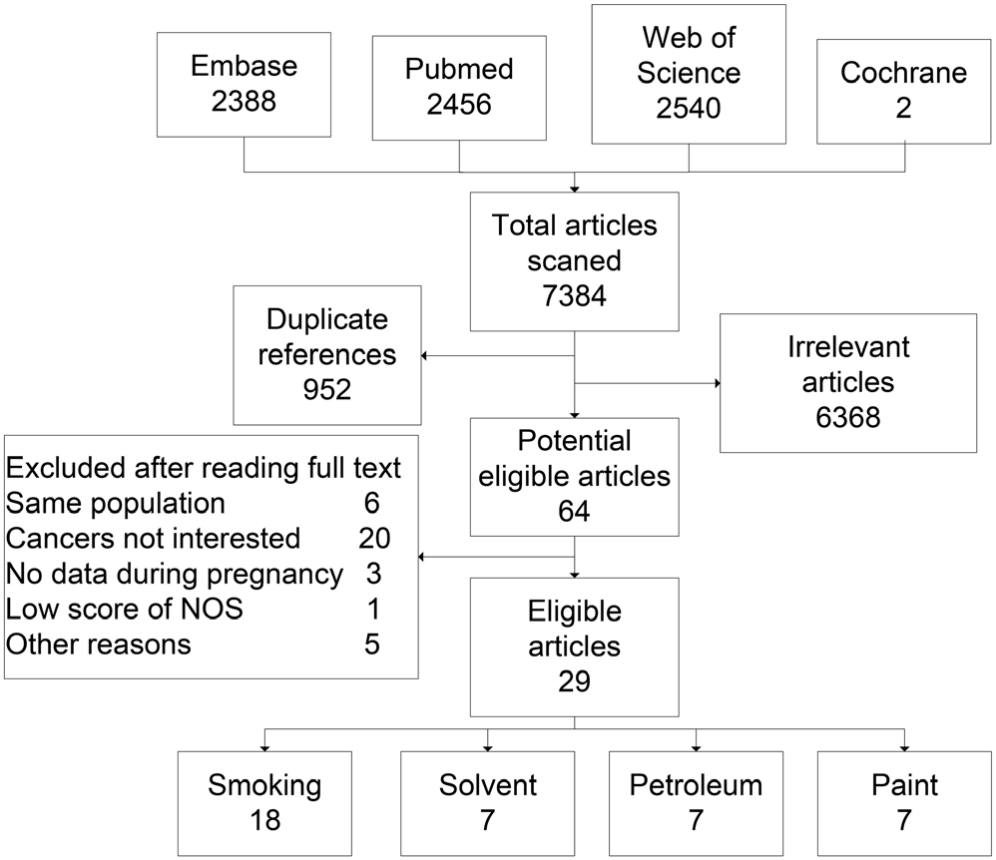
Literature search results. NOS, Newcastle-Ottawa-Scale.

The results of the search strategy and study selection process are presented in Figure 1. From a total of 7,384 studies identified, 952 were identified as duplicate studies and removed. By screening of titles or abstracts, 6,368 articles irrelevant to the study were excluded. After reading the full text, 20 studies were excluded as they only reported RR for all cancer or overall childhood leukemia. Six studies [30]–[35] were excluded because they used the same study population as one of the included studies. Five studies were excluded because they did not include data during pregnancy, one study was excluded due to low quality of NOS [36]. We also searched the reference lists of retrieved original articles and recent reviews, no additional studies were found. A total of 29 studies, including twenty eight case-control studies and [1], [2], [18], [37]–[61] one cohort study [62] were analyzed for this paper.

### Study characteristics

Table 2 and Table 3 summarized the main characteristics of the selected studies for analysis. Among the twenty-nine eligible studies, eleven studies were conducted in North America, three studies in the United Kingdom (UK), three studies in France, two studies in Latin America, two studies in Italy, and two studies in Australia. Other six studies were conducted in Germany, Sweden, Israel, Spain, Greece, United States, respectively Across the studies, twenty-seven reported OR, one study reported RR [51], and one reported HR [62]. Children’s age across the studies was under 15 years of old, except for one study by Sorahan et al. which was under 16 years old. The study period ranged from one year to 20 years. The studies used a variety of factors to adjust their analyses including age, sex, birth weight, study region, maternal age/education/race, family income.

**Table 2:** Characteristics of Studies on the Association Between Maternal Solvent, Paint, Petroleum Exposure During Pregnancy and Risk of ALL.

Study	County	Study design	Study period	Age range	Matching factors	Adjusting factors	Case	Control	Assessment of exposure	NOS score	Exposure definition	Effect estimate (OR,RR,HR) with 95% CIs
Shu et al.[59]	US	Case-Control	1989–1993	<15	Age, race, telephone, area code, exchange	Maternal education, race and family income	1842	1986	Telephone interviews	7	Solvents	1.6(1.1–2.3)
											Paints or thinners	1.7(1.2–2.3)
											Petroleum products	0.9(0.5–1.5)
Schuz et al.[54]	German	Case-Control	1992–1997	<15	Gender, age, date of birth	Socio economic status, degree of urbanization	1138	2963	Telephone interviews	7	Solvents	1.3(0.8–1.9)
											Paints or lacquers	2.0(1.2–3.3)
											Oil products	1.6(0.8–2.9)
Infant-Rivardet al.[55]	Canada	Case-Control	1980–2000	<15	Age, sex	Maternal age and level of schooling	790	790	Telephone interviews	7	Solvents	1.00(0.78–1.28)
Infant-Rivardet al.[2]	Spain	Case-Control	1983–1985	<15	Year of birth, sex municipality	Birth year,sex place of residence	128	128	Personal interviews	7	Solvents	0.62(0.20–1.91)
											Oil or grease	0.50(0.09–2.73)
Mckinney et al.[56]	UK	Case-Control	1991–1996	0–14	Sex, age, residence	Age, sex, study region	1324	2633	Personal interviews	8	Solvents	1.5(1.1–2.0)
											Paints	0.9(0.6–1.4)
											Petrol	2.1(1.2–3.6)
Reid et al.[57]	Australia	Case-Control	2003–2006	<15	Age, sex, state of residence	Child sex, age, socioeconomic status, maternal smoking, drinking, age	379	854	Telephone interviews	8	Solvents	1.64(0.93–2.88)
											Paints	1.14(0.81–1.62)
Miligi et al.[58]	Italy	Case-control	1998–2001	0–10	Gender, age, residence	Gender, age and area	601	1044	Personal interviews	8	Solvents	1.1(0.7–1.8)
											Oils	1.1(0.5–2.6)
Scelo et al.[1]	US	Case-Control	1995–2005	<15	Date of birth, sex, Hispanic ethnicity, maternal race	Income	550	737	Personal interviews	6	Paints	1.21(0.88–1.67)
Freedman et al.[18]	Midwestern and mid-Atlantic states	Case-Control	1989–1993	0–14	Age, first 8 digits of the telephone number, race	Child’s age, sex, household income, maternal education	640	640	Personal interviews	6	Painter	1.1(0.9–1.5)
Slater et al.[47]	US, Canada	Case-control	1996–2006	<1	Age	Birth year, maternal age, race	264	324	Telephone interviews	6	Paint	1.02(0.72–1.44)
											Petroleum	1.60(0.90–2.83)
Castro-jimenez et al.[60]	Colombia	Case-Control	2000–2005	<15	Sex, age	Maternal age parental preconception smoking status and maternal socioeconomic status	85	85	Personal interviews	6	Oils	1.77(0.79–4.02)

Abbreviations: OR, odds ratio; RR, relative risk; HR, hazard ratio; CI, confidence interval; NOS, Newcastle-Ottawa-Scale; US, United States; USA,United States of America;UK, United Kingdom.

**Table 3:** Characteristics of Studies on the Association Between Maternal Smoking During Pregnancy and Risk of ALL.

Study	County	Study design	Study period	Age range	Matching factors	Adjusting factors	Case	Control	Assessment of exposure	NOS score	Maternal smoking definition	Effect estimate (OR,RR,HR) with 95% CIs
Metayer et al.[37]	USA	Case-Control	1995–2008	<15	Child’s age, hispanic ethnicity, maternal race	Household income, child’s age, sex, hispanic ethnicity, maternal race	767	975	Personal interview	7	Three monthbefore or during pregnancy	0.83(0.56–1.24)
MacArthur et al.[38]	Canada	Case-Control	1990–1994	0–14	Age, gender, area	Child’s ethnicity, residential mobility, annual household income, maternal education, maternal age at birth	351	399	Personal interview	7	Maternal smoking during pregnancy	1.25(0.89–1.77)
Magnani et al.[39]	Italy	Case-Control	1974–1984	0–14	Age, sex	Birth year of the child, maternal age, illness during pregnancy, socioeconomic factors	140	305	Personal interview	5	Maternal smoking during pregnancy	0.7(0.5–1.1)
Menegaux et al.[40]	France	Case-Control	1995–1998	<15	Age, gender, region	Age, gender, region, socio professional category, birth order	407	567	Self-administered questionaire	8	Maternal smoking during pregnancy	1.4(1–1.9)
Mucci et al.[62]	Sweden	Cohort	1983–1997	<15	None	Maternal age, education, birthplace, parity, birth year; baby’s gender, gestational age, birth weight	505	1440542	Personal interview	6	Maternal smoking during pregnancy	0.75(0.6–0.93)
Okcu et al.[41]	USA	Case-Control	1995	<5	Age, sex	Year of birth, sex, gestational age, maternal age, tobacco use, parity, race/ethnicity	79	2543	Ascertained from the birth certificates	5	Maternal smoking during pregnancy	1.13(0.55–2.33)^a^
Pang et al.[42]	UK	Case-Control	1991–1996	<15	Age, sex, geographical area	Parental age, deprivation	1449	7581	Personal interview	8	Maternal smoking during pregnancy	0.89(0.77–1.03)
Petridou et al.(updated) [43]	Greece	Case-Control	1996–2008	0–14	Age, sex	Birth weight, birth order, crowding index, maternal age at birth, education	720	720	Personal interview	6	Maternal smoking during pregnancy	1.19(0.84–1.6)
Rudant et al.[44]	France	Case-Control	2003–2004	<15	Age, sex	Age, gender, parental professional category, maternal age at the time of birth	647	1681	Telephone interview	7	Maternal smoking during pregnancy	1.2(0.9–1.5)
Ferreira et al.[45]	Brazil	Case-Control	1999–2007	<2	Age	Maternal age, education, oral contraceptives use, birth weight, skin color	193	423	Personal interview	5	Maternal smoking during pregnancy	0.94(0.70–1.27)^a^
Milne et al.[46]	Australia	Case-Control	2003–2007	<15	Age, sex, state of residence	Maternal age, birth order, parental education, birth defects, maternal alcohol consumption during pregnancy and child’s ethnicity	388	868	Self-administered questionaire	7	Maternal smoking during pregnancy	1.02(0.76–1.37)
Slater et al.[61]	US, Canada	Case-Control	1996–2006	<1	Child’s birth year	Age, education, race/ethnicity and alcohol use during pregnancy, household income and child’s age	264	324	Telephone interview	6	Maternal smoking during pregnancy	0.87(0.54–1.4)
Abadi-Korek et al.[48]	Israel	Case-Control	1984–2002	NA	Age, religion, gender	NA	112	112	Telephone interview	5	Maternal smoking during pregnancy	0.91(0.58–1.43)^a^
Menegaux et al.[49]	France	Case-Control	1995–1999	<15	Age, gender, center	Age, gender, center, origin	239	288	Personal interview	6	Maternal smoking during pregnancy	0.9(0.6–1.4)
Shu et al.[50]	US, Canada, Australia	Case-Control	1983–1988	<1.5	Age, telephone area, code, exchange, number	Sex, maternal age, education, alcohol consumption	203	558	Telephone interview	8	Maternal smoking during pregnancy	0.78(0.51–1.18)
Sorahan et al.[51]	UK	Case-Control	1953–1955	<16	Sex, date of birth	Other parent’s habits, age at birth, social class, sib ship position, obstetric radio-graph	367	367	Personal interview	6	Maternal smoking during pregnancy	1.24(1.01–1.52)
Brondum et al.[52]	North America	Case-Control	1989–1993	<15	Age, race, telephone area, code, exchange	Mother’s race and education	1842	1986	Telephone interview	7	Maternal smoking during pregnancy	1.06(0.91–1.23)
Chang et al.[53]	USA	Case–Control	1995–1997,1999–2002	<15	Age, sex, race/ethnicity	Household income	281	364	Personal interview	7	Maternal smoking during pregnancy	0.93(0.58–1.51)

Abbreviations: OR, odds ratio; RR, relative risk; HR, hazard ratio; CI, confidence interval; NOS, Newcastle-Ottawa-Scale; NA, not available; US, United States; USA,United States of America;UK, United Kingdom.

a Crude odds ratio was calculated from the data provided.

Based on the quality assessment of NOS, sixteen studies were in high quality (six studies scored 8 and ten studies scored 7) and thirteen were in moderate quality (nine studies scored 6 and four studies scored 5).

### Main analysis

**Figure 2:**
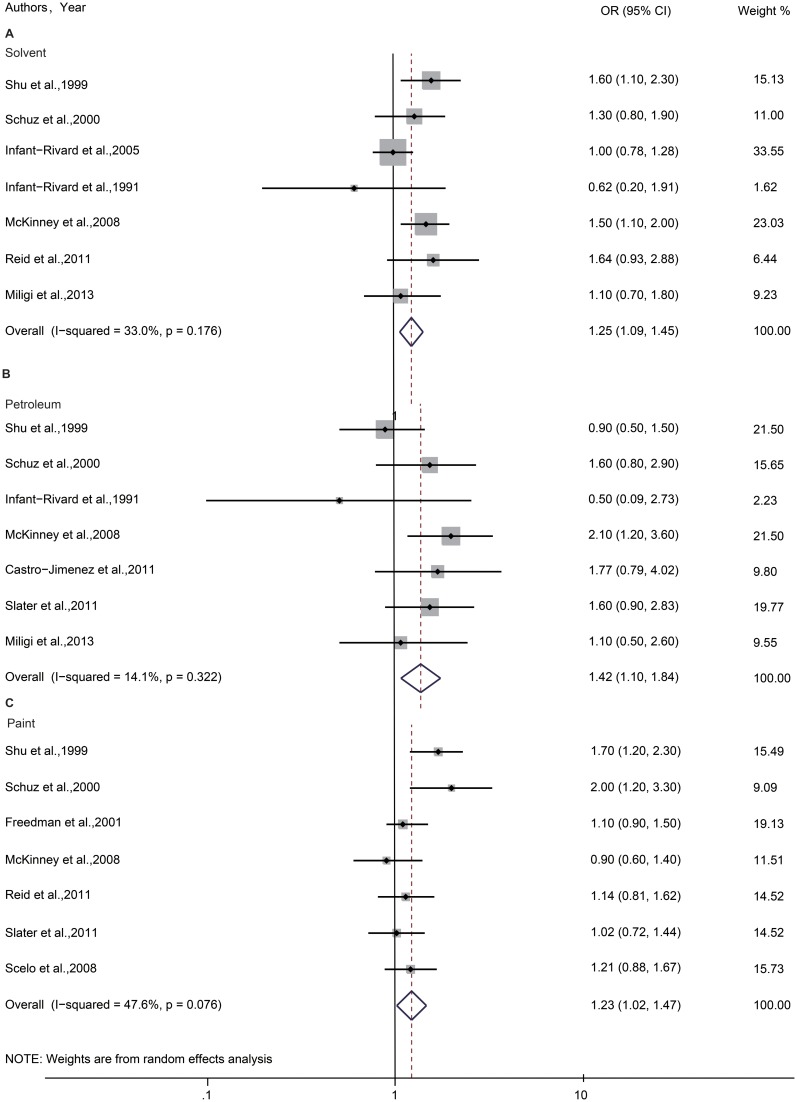
Forest plot of the association between maternal exposure during pregnancy and risk of childhood ALL. The size of each box indicates the relative weight of each study in the meta-analysis; the bars show the 95% confidence intervals (CIs). For paint, the weights are from random effects analysis because there are heterogeneity among studies (*P*<0.1).

As shown in Figure 2, no statistically significant heterogeneity across studies was found in solvent and petroleum exposure (*I^2^* = 33.0%, *P* = 0.176; *I^2^* = 14.1%, *P* = 0.322, respectively), however significant heterogeneity was observed in paint (*I^2^* = 47.6%, *P* = 0.076). The results in the meta-analysis showed an increased risk of ALL in those who were exposed to solvent or petroleum or paint (OR = 1.25, 95%CI = 1.09–1.45, fixed effect model; OR = 1.42, 95%CI = 1.10–1.84, fixed effect model; OR = 1.23, 95%CI = 1.02–1.47, random effect model, respectively).

**Figure 3:**
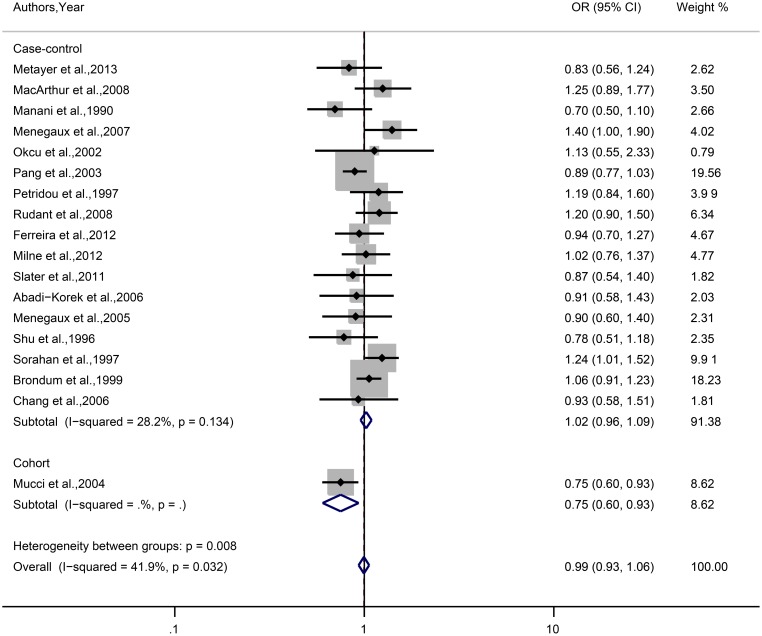
Forest plot of the association between maternal smoking during pregnancy and risk of childhood ALL. Considering study type may be the source of heterogeneity (*P* = 0.032), studies are divided into two subgroups (seventeen case-control studies (*P* = 0.134) and one cohort study). For studies of Abadi-Korek et al. [48] and Ferreira et al. [45], Crude odds ratios are calculated from the data provided.

Results for maternal smoking during pregnancy are shown in Figure 3. No significant associations were observed between maternal smoking during pregnancy and risk for childhood ALL (OR = 0.99, 95%CI = 0.93–1.06) in random effects model with heterogeneity (*I^2^* = 41.9%, *P* = 0.032) among these studies.

### Subgroup analysis, Sensitivity analysis and Publication bias

The results for subgroup analysis of childhood ALL incidence and maternal smoking during pregnancy are presented in Table 4. Consistent results were observed across the subgroup analyses including study period before or after 2000, sample size greater than or less than 1000, study region in Europe or other region, children’s age under five or not, study quality, maternal education, maternal age, household income, and ethnicity groups. In sensitivity analysis, after excluding the studies which potentially affected the pooled results no significant changes were observed (data not shown).

**Table 4:** Summary of the results for childhood ALL in relation to maternal smoking during pregnancy: subgroup analyses.

Factor	Level	No. of studies	%*I ^2^* (*P* value)^a^	OR(95%CI)	
				Fixed effects model	Random effects model
Total		18	41.9(0.032)	0.99(0.93,1.06)	0.99(0.91,1.09)
Study period	<2000^b^	12	55.3(0.001)	0.98(0.91,1.06)	0.98(0.86,1.11)
	≥2000^b^	6	0(0.533)	1.04(0.91,1.18)	1.04(0.91,1.18)
Sample size	<1000	10	39.5(0.094)	1.05(0.94,1.17)	1.01(0.87,1.17)
	≥1000	8	46.4(0.071)	0.97(0.89,1.15)	0.98(0.86,1.10)
Study region	Europe c	8	70.8(0.001)	0.99(0.91,1.08)	1.01(0.85,1.20)
	Other c	10	0(0.811)	1.00(0.91,1.11)	1.00(0.91,1.11)
Child’s age	<5	5	0(0.920)	0.90(0.75,1.09)	0.90(0.75,1.09)
	5–16	13	55.7(0.007)	1.01(0.94,1.08)	1.02(0.91,1.14)
Study quality	High d	9	38.5(0.112)	1.12(0.94,1.10)	1.03(0.92,1.16)
	Moderate^d^	9	48.7(0.049)	0.96(0.86,1.07)	0.95(0.81,1.11)
Maternal education	Adjusted	7	21.8(0.236)	1.03(0.94,1.13)	1.03(0.88,1.20)
	No	11	20.1(0.020)	0.96(0.87,1.05)	0.96(0.86,1.08)
Maternal age	Adjusted	10	55.9(0.015)	1.02(0.92,1.12)	1.00(0.86,1.17)
	No	8	17.9(0.289)	0.98(0.90,1.07)	0.98(0.88,1.19)
Household income	Adjusted	7	47.9(0.079)	1.07(0.93,1.22)	1.03(0.85,1.25)
	No	11	39.8(0.083)	0.97(0.91,1.05)	0.97(0.88,1.08)
Ethnicity	Adjusted	5	0(0.561)	1.05(0.92,1.18)	1.05(0.92,1.18)
	No	13	52.8(0.013)	0.98(0.91,1.05)	0.98(0.87,1.11)

Abbreviations: OR, odds ratio.

a Higgins’ *I^2^* statistic and 95%CI, are shown for a measure of the degree of heterogeneity across studies.

b <2000 means before the year of 2000, ≥2000 means after the year 2000.

c Europe counties include Italy, France, Sweden, United Kingdom, Greece. Others include America, Canada, Australia, Israel, Brazil.

d “high” means the score of quality range from 7 to 9, “moderate” means the score is among 4 to 6.

**Figure 4:**
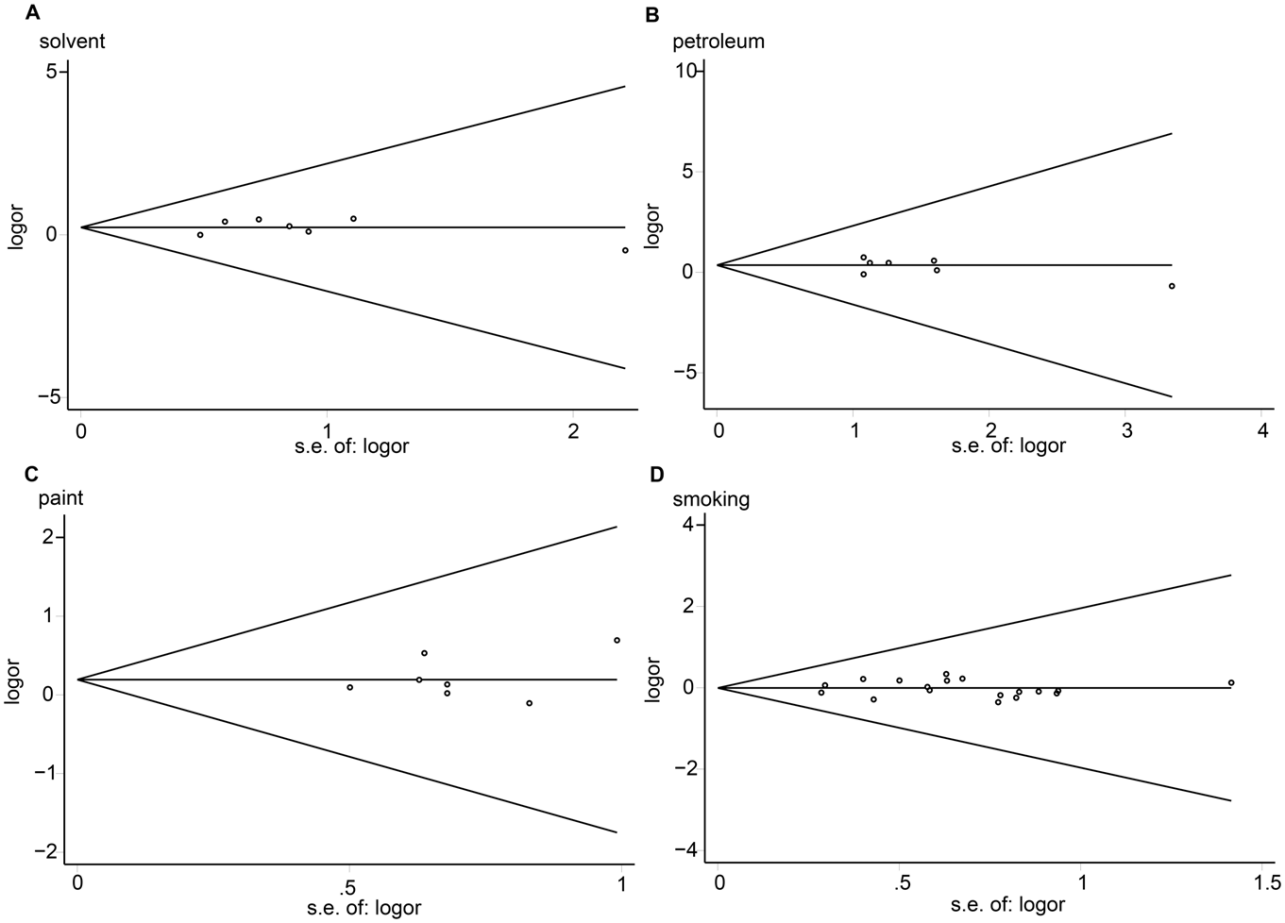
Funnel plot of the association between maternal exposure and the risk of childhood ALL.

Using Egger’s and Begg’s test, no publication bias was observed in this meta-analysis (seen in Figure 4), for solvent (Egger’s test: *P* = 0.996; Begg’s test: *P* = 0.764), paint (Egger’s test: *P* = 0.533; Begg’s test: *P* = 0.879), petroleum (Egger’s test: *P* = 0.378; Begg’s test: *P* = 0.448), and smoking (Egger’s test: *P* = 0.836; Begg’s test: *P* = 0.649).

## Discussion

Our meta-analysis included data from 29 studies with 16,695 cases and 1,472,786 controls. Based on the present meta-analysis, the overall data demonstrated that the ALL was significantly associated with maternal solvent, paint, or petroleum exposure. For maternal smoking during pregnancy, the same conclusion has been concluded for risk of childhood ALL in previous meta-analysis (OR = 1.03, 95% CI: 0.95–1.12) [13]. However, Different with the previous meta-analysis which only examined the association between maternal smoking and childhood ALL, this study focused the exposure of solvent, paint and petroleum exposure for children ALL. In addition, we updated the literatures in this meta-analysis, assessed the study quality we analyzed, and performed subgroup analysis in this paper.

Exposures to solvent and paint have been suggested as potential risk factors of childhood leukemia [1]. A study of cancer patients in Montréal, Canada estimated that 40% of workers had been occupationally exposed to at least one solvent over their careers [55]. Another study indicated that home exposure to solvents might also be associated with childhood ALL [18]. However, one challenge for the study with parental occupation and childhood ALL is that maternal exposure during pregnancy was rarely assessed [57], and the exposure strength at home is low. Mothers who worked as a painter or were exposed to paints were consistently associated with childhood leukemia [57]. Paint consists of various compositions, but we are not sure whether they influence the identified genetic “hits” or influence the progression of the disease via other means. The toxicity of maternal paint intake may differ according to the kinds of paints. One of the two major groups of paints is latex paints, in which the solvent is water. The other is the alkyd paints or oil-based resin paints, in which the solvent is usually petroleum-based and organic, such as toluene or xylene [1]. Nevertheless, avoiding the use of paint in the house during pregnancy and early childhood would be a prudent measure [1].

Petroleum is another main source of environmental benzene exposure. Studies have shown that childhood leukemia is associated with various exposures to petroleum. A study by Swaen et al. indicated leukemia mortality was associated with gasoline consumption, number of household cars, distance to a gasoline station, and distance to main road as evaluation indexes of petroleum [63]. Considering that the exposure dose among children is difficult to assess, in this meta-analysis, we restricted maternal occupational exposure to petroleum products as the index for children leukemia.

Several studies specifically evaluated childhood ALL risk associated with maternal smoking during pregnancy. However, only three case–control studies [36], [49], [51] reported significant association for children ALL risk (OR: 2.20, 1.40 and 1.24, respectively). In addition, the sole cohort study [62] conducted in Sweden reported a non-statistically significant association in the same population at a later time period than the preceding case–control study [36], with the proportion of maternal heavy smoking during pregnant three times lower than that reported in the earlier Swedish study [36]. Additionally, there are also studies suggesting that tobacco exposure induces in vivo fragile-site expression, which contributes to tumor formation [64]. Although non-statistically significant associations were shown between maternal smoking during pregnancy and ALL in this meta-analysis, parental cigarette smoking should also be strictly prohibited. It is suggested that parental smoking affects many childhood diseases, such as respiratory tract infection, asthma and otitis media, which are much more prevalent than childhood leukemia [45].

To detect the source of heterogeneity, we conducted subgroup analysis by region, year, child’s age, sample size of each study, high or moderate study quality and whether the effect size had adjusted by maternal education, maternal age, household income or ethnicity groups. Both fixed and random effect model were used for each subgroup. No heterogeneity was found across these subgroup variables, and the result of each subgroup was similar to the overall effect size. However, we did find the heterogeneity for types of study design and low quality study in our preliminary analysis. Therefore, we analyzed case-control studies and cohort studies separately and excluded the one low quality study and no heterogeneity was observed in this meta-analysis.

Our study may have the following limitations. First, interviewer bias: 1) The data in most studies we analyzed were collected by personal interview [1], [2], [18], [37]–[39], [42], [43], [45], [49], [51], [53], [56], [58], [60], [62] or independent telephone interviews [44], [47], [48], [50], [52], [54], [55], [57], [59], [61]. Several studies’ data were collected by self-administered questionnaire [40], [46] or ascertained from the birth certificates [41]. The participants’ attitudes and their understanding of the questions may have differed across populations [65]; 2) interviewers could not be blinded to case-control status, one study used different interviewers for case and control parents [51]. Second, recall bias: 1) parents were asked for the exposure occurred many years ago, which may not be remembered clearly; 2) parents who had a child with leukemia might tend to recall greater or perhaps distorted levels of exposure. Furthermore, we only collected articles published in English, which could bring publication bias, despite there being no significant evidence of publication bias observed using Egger’s test or Begg’s test. In addition, not all of the studies adjusted for family income, while socioeconomic status has been associated with the risk of childhood leukemia [66]. For example, indoor house painting was more common among high-income controls, which suggests that a selection bias. Finally, study design might have influenced the results since only one cohort study was included.

Despite the limitations, there are several strengths of this study. First, twenty-nine studies cover a broad region and long study period. Second, all the eligible studies had high or moderate research design quality. Third, no publication bias was observed based on Egger’s test and Begg’s test. Finally, our eligible literatures had comprehensive matching factors and adjusting factors, thus reducing the corresponding errors.

## Conclusion

Based on the present meta-analysis, we concluded that maternal solvent, paint, petroleum exposure during pregnancy are associated with ALL. Avoidance of maternal benzene exposure during pregnancy might contribute to a decrease in the risk of childhood ALL.

This meta-analysis suggested the directions for future study are: 1) conduct large sample cohort studies or high quality case–control studies; 2) investigate new risk factors for childhood leukemia, such as drug use, traffic exhaust, alcohol consumption, infection, etc.; 3) explore the exposure during pregnancy, as well as before pregnancy and childhood exposure; 4) explore dose-response relationships; 5) assess gene-environment interaction, as the mechanism of childhood leukemia is likely to associate with gene–environment interactions [67].

## Supporting Information

Checklist S1 **PRISMA 2009 Checklist.**
(DOC)
